# Modified superior oblique intrasheath tenectomy in A-pattern with superior oblique overaction

**DOI:** 10.1186/s12886-021-01942-2

**Published:** 2021-04-21

**Authors:** Chunhua Sun, Ze Wang, Bo Xia

**Affiliations:** 1 Tianjin Eye Hospital, Clinical College of Ophthalmology of Tianjin Medical University, Nankai University Affiliated Eye Hospital, Tianjin Key Laboratory of Ophthalmology and Visual Science, Tianjin Eye Institute, 4 Gansu Road, 300020 Tianjin, China; 2 Nanjing South East Eye Hospital, 36 Muxuyuan Street, 210007 Nanjing, China

**Keywords:** Intrasheath tenectomy, Superior oblique overaction, A-pattern, Minimal incision, Operating microscope, Traction test

## Abstract

**Background:**

To evaluate the effect of modified superior oblique intrasheath tenectomy (MSOIT) on superior oblique overaction (SOOA) with A-pattern.

**Methods:**

We retrospectively reviewed the data of 66 patients (130 eyes) with SOOA and A-pattern underwent MSOIT at the nasal border of the superior rectus under an operating microscope between January 1, 2004 and December 31, 2018. The superior oblique (SO) tendon fibres were resected, and the sheath was preserved in all patients. The preoperative and postoperative SOOA, objective torsion, ocular motility, and A-pattern deviation findings were compared. The correlation between the preoperative A-pattern deviation and the corrected deviation was analysed. The average follow-up period was 33.45 ± 29.88 (range: 12–122) months.

**Results:**

The mean SOOA deviation improved from 2.95 ± 0.54 to 0.34 ± 0.55 (*P* < 0.001), while the A-pattern deviation difference between upgaze and downgaze improved from 23.15 ± 7.59 prism diopters (PD) to 3.50 ± 2.90 PD (*P* < 0.001). The average objective fundus intorsion value improved from + 2.96 ± 0.58 to + 0.38 ± 0.60 (*P* < 0.001). The magnitude of correction in A-pattern was significantly correlated with the preoperative severity of A-pattern (*r* = 0.812, *P* < 0.001).

**Conclusions:**

MSOIT at the nasal border of the superior rectus (SR) under an operating microscope is safe and yields beneficial outcomes in patients with SOOA and A-pattern.

## Background

Superior oblique overaction (SOOA) corresponds to excessive activity of the superior oblique muscle in directions including intorsion, depression, and abduction [1]. Patients with SOOA may show abduction in downgaze, causing A-pattern [1]. The superior oblique (SO) weakening procedures include tenotomy, tenectomy, recession, split-tendon lengthening, and silicone tendon expander [1]. Tendon scarring due to split-tendon lengthening, a new insertion limiting SO depression due to recession, uncontrolled effects caused by tenotomy, and the risk of the infection and silicone sticking to the sclera by the silicone expander are severe complications that increase the complexity of strabismus conditions [1–6]. The frenulum between the SO and superior rectus (SR) also limits the effects of disinsertion and suspension recession with a temporal approach [5, 6]. SO tenotomy with preservation of the fascia in the vicinity the tendon achieves more predictable results [7]. Intrasheath tenotomy or tenectomy of the SO tendon is advantageous because it prevents SO paralysis and achieves more sufficient weakening because the cut ends remain within the sheath [8]. Under a microscope, minimal anatomical disruption, swelling and pain and good visualization have been achieved in rectus muscle surgery and oblique muscle recession [9]. Over the past 15 years, we have performed modified superior oblique intrasheath tenectomy (MSOIT) with a small incision under a surgical microscope and found that it is effective for treating A-pattern with SOOA.

## Methods

### Patients

A total of 66 patients (32 men, 34 women; average age: 19.38 ± 11.67 years) underwent MSOIT with or without horizontal rectus operations in 130 eyes between January 2004 and December 2018, and patients who exhibited A-pattern with SOOA and were followed up for more than 1 year were considered eligible. None of the patients exhibited stereopsis with less than 3000 s of arc with the Titmus stereo test.

The study protocol adhered to the tenets of the Declaration of Helsinki. Informed consent for the procedure and the present study was provided by the patients or by the parents or the legal guardians of children. All procedures were supervised by Dr. Wang. The preoperative and postoperative measurements were compared. SO dysfunction was measured with a scale of -4 to + 4 with 0 indicating normal and + 4 indicating maximum overaction [1]. A measurement of + 1 was recorded if there was no hypotropia with horizontal versions but there was slight overaction when the eye was moved into the field of action of the SO vertically. Slight hypotropia in horizontal gaze was recorded as ‘+2’, while ‘+3’ indicated obvious hypotropia in direct horizontal gaze, and ‘+4’ indicated large hypotropia in horizontal gaze with an abduction movement as the eye moved vertically into its field of action. Objective torsion was graded on a scale of 0–4 based on the fundus photographs [10]. Deviation was measured with an alternate prism and cover test while the patient fixated at a distance of 6 m in a 25° upgaze, the primary position, and a 25° downgaze. The difference in horizontal deviation between the upgaze and downgaze conditions was recorded. A-pattern was diagnosed as greater divergence in downgaze versus upgaze of at least 10 prism diopters. All patients underwent assessment before surgery and were asked to undergo postoperative follow-up assessments at 1, 3, and 6 months and every 6 months thereafter. Adverse effects were recorded. The patients’ data were analysed using SPSS 17.0 statistical software (SPSS Inc., Chicago, IL, USA), and comparisons were made between the preoperative and 12-month postoperative data. The average follow-up period was 33.45 ± 29.88 (range: 12–122) months. Wilcoxon’s test was applied for statistical analysis. Spearman’s correlation analysis was used to analyse the correlation between the preoperative A-pattern deviation and corrected deviation.

### MSOIT procedure

**Fig. 1:**
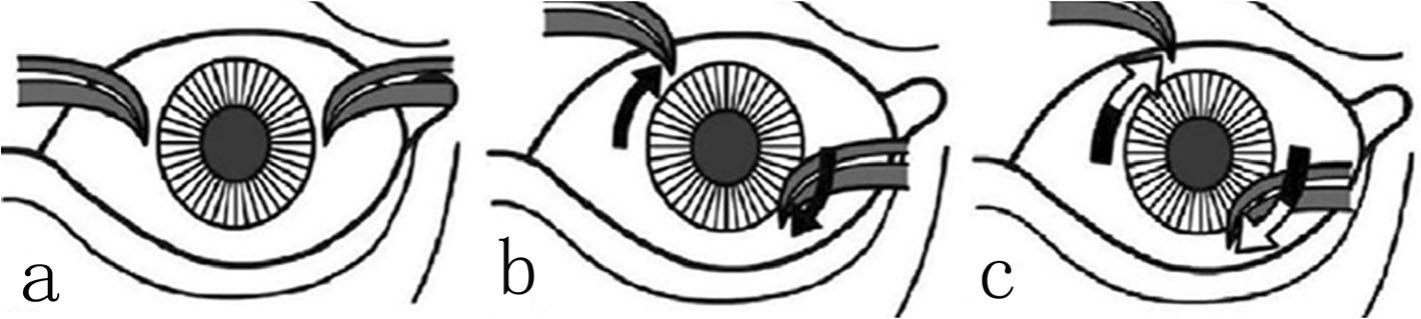
Simplified forced extorsion traction test. **a**: The left eye is grasped and retroplaced at the 3 and 9 o’clock positions on the limbus. **b**: The eye is rotated clockwise. In the case of superior oblique overaction (SOOA), the forceps, located at the 3 o’clock position, could not be extorted to the 5 o’clock position before surgery. **c**: After surgery, eye extorsion of an additional one to one-and-a-half clock hours could be obtained.

**Fig. 2:**
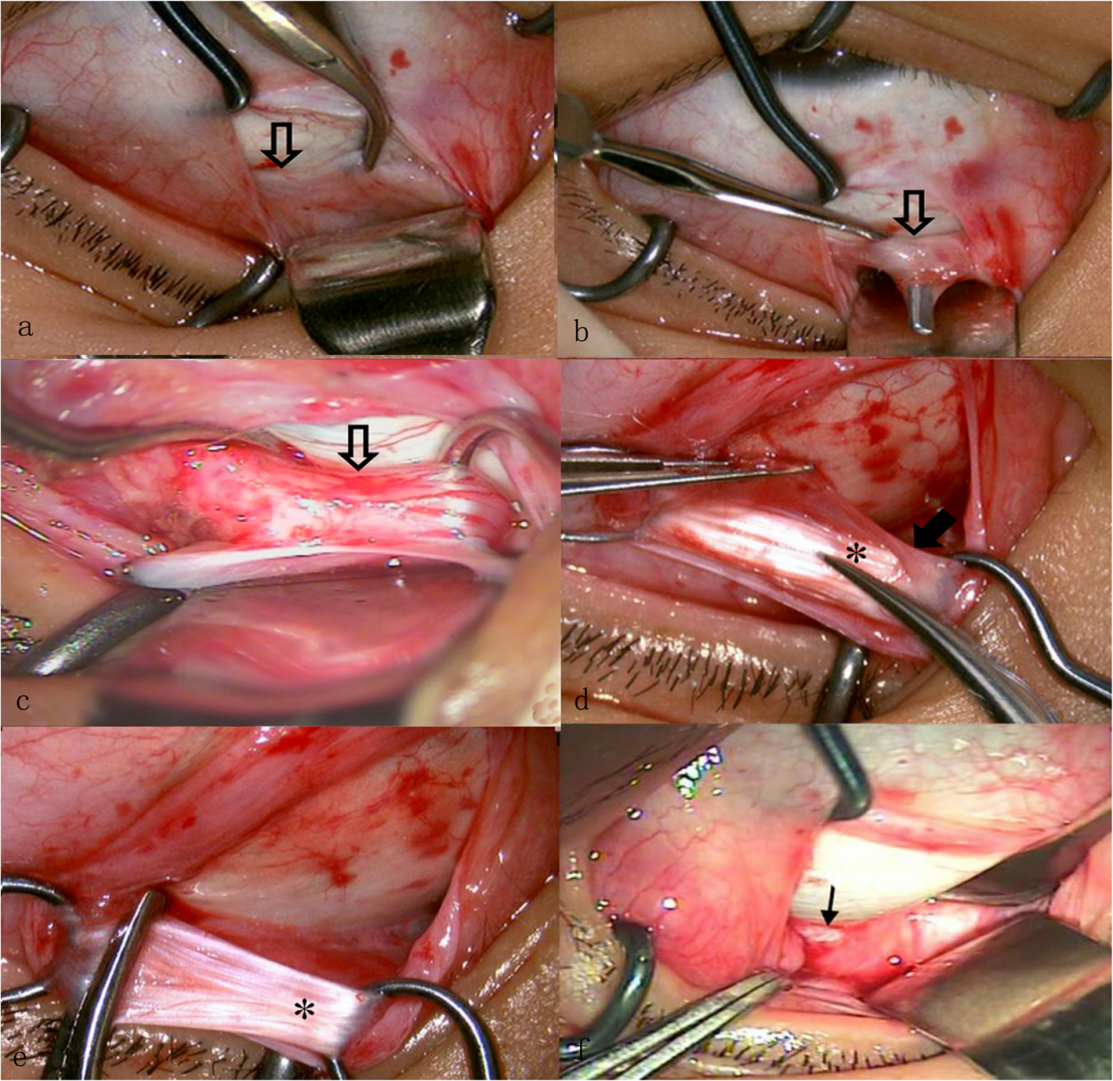
Superior oblique (SO) tendon and sheath. **a**: The SO was identified by its pearly white glistening appearance against the sclera. **b**: The SO was elevated with small hook. **c**: Two ancistroid hooks were used to fix the SO muscle at the two borders. Blood vessels were detected along the sheath in some cases. **d**: Solid arrow: The SO tendon is bluntly isolated from the sheath. **e**: The naked SO tendon is held with two ancistroid hooks at the two borders. The temporal border of the naked tendon is clamped. **f**: Small disturbed area is visible after surgery using an operating microscope (black arrow). Hollow arrow: SO; solid arrow: SO sheath; stars: SO tendon; thin arrow: small area of disturbance of the fascial tissues.

The traction test was performed before and after the procedure with a simplification of Guyton’s exaggerated traction test [11] (Fig. 1). The globe was grasped and retro-placed using 0.5-mm toothed forceps at the 3 and 9 o’clock positions on the limbus. Then, the right eye was rotated counterclockwise, and the left eye was rotated clockwise. The eye was subjected to resistance but was not extorted by more than two clock hours. The operating microscope magnification was set to 4×. A super-nasal fornix incision was made 6–8 mm posterior to the limbus. Separate incisions in Tenon’s capsule and the conjunctiva were completed at right angles to expose the sclera at the nasal border of the SR. The SR and Tenon’s capsule were hooked and then moved inferior-temporally. A Desmarres retractor was placed at the posterior aspect of the incision and pulled backward to adequately visualize the triangular area formed by the SO and the nasal border of the SR. In cases in which exposure was limited, a set of custom-made deep retractors, similar to Desmarres retractor except with longer folds of 5 mm, 10 mm, 15 mm, and 20 mm, were applied progressively until the SO tendon was located. The SO tendon was identified by its pearly white glistening appearance, running perpendicular to the SR against the sclera (Fig. 2a). The SO was elevated with the small hook (Fig. 2b) and hooked by two ancistroid hooks to prevent slipping. In some cases, blood vessels were observed along the sheath (Fig. 2c). A higher magnification (6× to 8×) was used for the subsequent procedures. A 6–8 mm incision in the long axis of the anterior aspect of the sheath was made to expose the tendon fibres, and the blood vessels were avoided. Six millimetres of tendon fibres were bluntly separated from the sheath (Fig. 2d and e), and the naked tendon was resected in its sheath. With a special deep retractor (20 mm), we inspected and verified that no posterior tendon fibres remained. Following the procedure, only a small area of disturbance was visible in the fascial tissues (Fig. 2f). To ensure that the tendon had been completely transected, the simplified forced extorsion traction test was repeated, additional extorsion of one to one-and-a-half clock hours was achieved, and the eyeball felt “softer”.

## Results

**Fig. 3:**
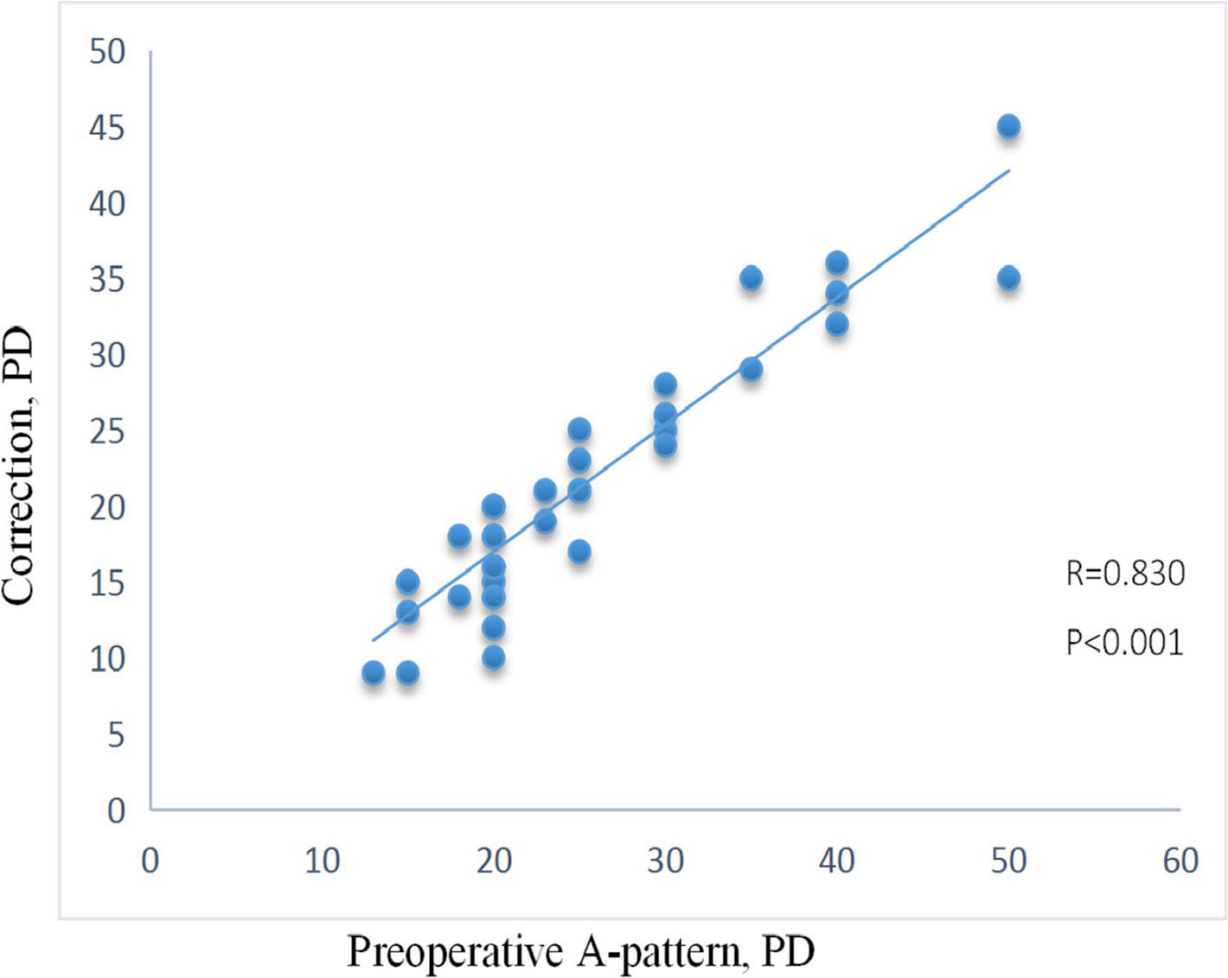
Scatterplot of preoperative A-pattern deviation and correction. PD, prism diopters.

In all 130 eyes, the SO tendon and sheath were identified and operated on successfully without any adverse effects. Only one eye suffered from mild inferior oblique overaction without obvious superior oblique underaction in our early experience. Blood vessels were encountered along the sheath in some eyes [12]. The differences in SOOA severity, horizontal deviation, and intorsion were inconsistent; therefore, Wilcoxon’s test was applied (Table 1). The magnitude of correction of A-pattern was significantly correlated with the preoperative severity of A-pattern (Fig. 3, *r* = 0.830, *P* < 0.001). None of the patients showed poorer results in these parameters at 12 months postoperatively.

**Table 1:** Results of superior oblique muscle intrasheath tenectomy

	Before operation	After operation	*Z*	*P*
^a^SOOA (+)	2.95 ± 0.54	0.34 ± 0.55	-10.12	0.000
Objective intorsion (+)	2.96 ± 0.58	0.38 ± 0.60	-10.07	0.000
^b^A pattern deviation (PD)	23.15 ± 7.59	3.50 ± 2.90	-7.07	0.000

^a^*SOOA* superior oblique overaction; ^b^ Difference in horizontal deviation between the upgaze and downgaze conditions in A-pattern; *PD* prism diopters

## Discussion

### The simplified forced torsion traction test

Guyton’s exaggerated traction test is considered invaluable for assessing the tightness of the oblique [6]. Jung and Holmes proposed a new traction test in which the globe is maximally excyclorotated without retroplacement until the first instance of resistance is felt [13]. The authors reported a median maximum excyclorotation of 62.5 degrees after SO disinsertion in six eyes. In the current simplified forced torsion traction test, the eyeball is grasped and retro-pulsed, thereby stretching the oblique muscles and adding slack to the rectus muscles. Compared with the reported SO forced traction test [3, 4, 6, 11, 13] used in SO-weakening procedures, the present test is simpler to perform. It is practical to evaluate torsion traction tests by the number of hours required in an operation, especially for inexperienced surgeons. Residual SOOA is likely the most common complication of SO-weakening procedures, which results in a positive exaggerated forced duction test result [3]. If the tenectomy is completed, additional extorsion of one or one-and-a-half clock hours can be achieved; if not, the surgeon needs to inspect and verify whether the tendon fibres have been severed completely.

### Minimal disturbance with visualization under a microscope

Both the complexity of the function and anatomical variation in the SO lead to challenges in isolating and manipulating the SO during the procedure [8, 14]. Without visualization, SO tendon isolation can lead to injury of the vortex vein and orbital fat prolapse, a posterior Tenon’s capsule tear, iatrogenic ptosis, the SR being mistaken for the SO, and failure to hook the SO tendon [15]. Excessively dissecting Tenon’s capsule overlying the tendon may result in postoperative restricted globe elevation and even acquired Brown syndrome [14]. A preferred nasal-weakening procedure with a temporal conjunctival incision could keep the nasal intermuscular septum intact and reduce the risk for postoperative SO palsy or limited depression [3]. In the present study, the nasal approach was used, and a small incision with minimal disturbance was made under an operating microscope. Low-power magnification (4×) is required for SO tendon identification. The distance between the anterior border of the SO and the SR insertion varies from 3 to 4 mm to 8 mm with the eye moving from primary to downward position [15], which make it difficult to identify the SO. With a sufficient operating field and appropriate depth, this difficulty can be overcome by using different retractors with increasing lengths to 20 mm for individual patients. A sheath was detected to be enveloping the tendon (Fig. 2d) in all cases, which is consistent with the findings of Helveston’s study [16]. Blood vessels were noted along the sheath in some cases (Fig. 2c) [12] and were preserved with intrasheath tenectomy under a microscope, which is inconsistent with the findings of previous reports [15]. With a higher magnification, at 6× to 8×, a small incision in the intermuscular septum and sheath was sufficient to explore the tendon fibres while keeping the deeper intermuscular septum intact (Fig. 2e and e), thereby avoiding the surrounding adhesion.

### Intrasheath tenectomy at the nasal border of the SR

SO tenotomy can also be performed from the temporal side of the SR [14], but the weakening effect is smaller than that with nasal tenotomy [3, 4]. Wei et al. reported that bilateral SO posterior tenectomy is effective for treating mild and moderate SOOA-associated A-pattern [17]. The mean SOOA value decreased by 1.85, with a mean preoperative A-pattern deviation reduction of 12.75 PD, which were smaller than the values in the present study. The closer to the trochlea that tenotomy is performed, the more effective the procedure is [1, 3, 14]. Furthermore, the ‘frenulum’ between the SO and SR can hinder the SO weakening effect of temporal disinsertion or suspension recession [5]. Debert et al. performed 6 mm bilateral SO transection with the complete width from its insertion without frenulum dissection [18]. The mean A-pattern collapse value was 18 PD, and the mean preoperative A-pattern deviation was 21 PD, which was similar to our result. However, four patients had postoperative vertical deviation. Heo et al. performed 10 mm SO posterior tenectomy with dissection of the frenulum to the extent possible [19]. The mean A-pattern correction was 17.63 PD, which was similar to that in our study. However, 5 of 75 patients showed mild inferior oblique overaction.

Among the controlled weakening procedures, Pollard reported the collapse of a 7 mm silicone tendon expander to 36.3 PD from 39.6 PD, which was larger than our result [20]. However, silicone expanders cause more severe inflammatory reactions as a foreign material, as well as a greater risk of adhesion syndrome [6, 14, 21]. Li reported hang-back recession of the SO with a mean horizontal deviation correction of A-pattern of 24.10 PD, which was a little greater than that in the current study, and their average A-pattern horizontal deviation before surgery (27.58 PD) was also more severe than ours [22].

Parks and colleagues performed intrasheath tenotomy or tenectomy to achieve a sufficient weakening effect [8]. Helveston [7] also proposed that the fascia around the SO tendon should be left undisturbed to achieve more predictable results. In the current study, MSOIT at the nasal border of the SR was performed for 130 eyes in 66 A-pattern patients with SOOA who were followed up for a mean of 33.45 months. Our inadequate early experience disturbing the fascia might be the reason for only mild inferior oblique overaction in one eye. Dissecting the sheath is a delicate task, which suggests surgeon’s experience is necessary. This treatment improved the mean A-pattern deviation by 19.65 PD, and the mean SOOA value improved by 2.61 (Table 1). The magnitude of correction of A-pattern was significantly correlated with the preoperative severity of A-pattern (Fig. 3). The more severe the preoperative A-pattern was, the greater the amount of weakness the MSOIT achieved. The SO tendon was weakened with minimal sheath disturbance and the frenulum left intact. The preserved sheath was still connected to the two cut tendon edges in its original course, causing minimal disturbance to the fascia and allowing connections to form. Due to the temporal section and insertion being left intact, nasal transection resolved the case of SOOA, preserved important function and avoided SO palsy [3].

## Limitations

The study included 66 patients with at least 12 months of follow-up data. Studies with larger sample sizes are needed. The study was retrospective and not controlled.

## Conclusions

MSOIT at the nasal border of the SR under an operating microscope is a feasible treatment for A-pattern caused by SOOA with minimal disturbance.

## Data Availability

The datasets used and/or analysed during the current study are available from the corresponding author on reasonable request.

## Abbreviations

MSOIT: Modified superior oblique intrasheath tenectomy
SOOA: Superior oblique overaction
SO: Superior oblique
PD: Prism diopters
SR: Superior rectus
